# Biochemical Correlates of Video Game Use: From Physiology to Pathology. A Narrative Review

**DOI:** 10.3390/life11080775

**Published:** 2021-07-30

**Authors:** Barbara Carpita, Dario Muti, Benedetta Nardi, Francesca Benedetti, Andrea Cappelli, Ivan Mirko Cremone, Claudia Carmassi, Liliana Dell’Osso

**Affiliations:** Department of Clinical and Experimental Medicine, University of Pisa, 56126 Pisa, Italy; benedetta.nardi@live.it (B.N.); francesca.benedetti1793@gmail.com (F.B.); andrea.cappelli1990@gmail.com (A.C.); ivan.cremone@gmail.com (I.M.C.); ccarmassi@gmail.com (C.C.); liliana.dellosso@med.unipi.it (L.D.)

**Keywords:** video games, Internet gaming disorders, neurobiology, biochemical correlates, biological psychiatry

## Abstract

In the last few decades, video game playing progressively became a widespread activity for many people, in childhood as well in adulthood. An increasing amount of literature has focused on pathological and non-pathological correlates of video game playing, with specific attention towards Internet Gaming Disorder (IGD). While many neurobiological studies in this field were based on neuroimaging, highlighting structural and functional brain changes among video game users, only a limited number of studies investigated the presence of biochemical correlates of video gaming. The present work aims to summarize and review the available literature about biochemical changes linked to video game use in IGD patients as well as non-pathological users, and the differences in between. Results may shed light on risks and benefits of video games, providing directions for further research on IGD treatment and, on other hand, on the potential role of video games in therapeutic or preventive protocols for specific conditions.

## 1. Introduction

Internet gaming disorder (IGD) has represented a matter of interest in psychiatric research since the early 1990s. It is usually defined as a continuous and repeated involvement with video games, often leading to significant daily, work, and/or educational disruptions [1,2,3]. The first report of a pathological video game habits, described by Keepers [4] in a young boy, and other subsequent similar descriptions, progressively led to a hypothesis that video games and the Internet may actually be associated, in some subjects, with addiction or dependence-related problems. Several labels have been used to name this pathological behaviour, without a common agreement, and including or not the problematic use of video games outside the Internet [5]. Until the last few years, IGD was called as problem video game use, video game addiction, computer addiction, compulsive Internet use, pathological Internet use, maladaptive Internet use, Internet addiction, problematic Internet use, and virtual addiction [5]. To date, IGD is not included in the most recent version of the Diagnostic and Statistical Manual of Mental Disorders (DSM-5) [1], but it figures in Section 3 of the manual for conditions that require further studies. IGD has been considered as a behavioural addiction and several common features were highlighted between IGD, gambling, and substance use disorders (SUD), such as self-directedness and high neuroticism [6,7].

Several psychiatric conditions, such as impulsiveness, depression, social anxiety, obsessive-compulsive disorder (OCD), and autism spectrum disorder (ASD) have been more frequently associated with the presence of IGD [8,9,10]. Other authors showed strong association of IGD with Hikikomori Syndrome, a severe form of social withdrawal, suggesting bidirectional interactions between these two conditions [11]. However, previous studies in this field did not clarify if IGD should be considered, with respect to the other disorders in comorbidity, as a predisposing factor, as a consequence, or simply as a co-occurring condition. In this latter case, it is possible that IGD would share some vulnerability factors with other kinds of mental disorders [8,9,10,11].

According to previous studies, common neuroimaging alterations were reported between IGD and SUD, including decreased gray matter volume and lower density in the anterior cingulate cortex, an increased gray matter volume in striatal regions [12], or an increased reactivity of the striatum [13]. However, less is known about the specific neurobiological mechanisms underlying IGD. According to some authors [12], IGD may imply the release of strong dopaminergic bursts from striatal regions, which may feature functional changes in the dorsolateral prefrontal cortex, resulting in a reduction of the executive functions, with a potential gradual loss of control on gaming use. An important role would also be played by the mesolimbic dopamine system [14] in the development and maintenance of the pathological habit, as observed in other behavioural or substance addictions. Park et al. [15], through the observation of resting-state PET scans, found a reduced metabolism in the precentral gyrus of IGD subjects, suggesting an insensitivity to negative consequences of excessive game play, and an increased metabolism in the middle orbitofrontal gyrus, which may reflect compensatory cognitive processing. The corpus callous also represents an important structure showing alterations in IGD [16]. A structural and functional impairment of the corpus callosum, frequently observed in subjects affected by SUD [17], was described in IGD individuals as a reduced fractional anisotropy of this brain structure [16]. Several studies also reported altered physiological reactions of cardiovascular and respiratory systems in video game users [18,19]. Cardiovascular reactivity to video games in young men was also considered as a predictor for hypertension [20]. On the other hand, some studies observed how the experience of playing electronic games can produce several positive effects on human health, although these effects depend on the type of video game [21]. Some kinds of video games may also be used for didactic or therapeutic purposes in different fields, such as training physical abilities or interpersonal skills [22,23,24]. Furthermore, previous studies found a correlation between video game training and cognitive enhancements [25,26] in terms of working memory performance, task-related cortical activity [27,28], time reaction [29], short-term memory, and visual attention [30].

Despite the increasing body of neuroimaging and neurophysiological studies about the effects of video game playing among IGD patients and, to a lesser extent, among non-pathological video game users, to date, only a limited amount of research specifically investigated the biochemical correlates of video game use. Most of the biochemical studies in this field seem to confirm the central role of dopamine (DA) in this condition, a neurotransmitter already known for its crucial role in drug and alcohol dependence, mediating reward and withdrawal mechanisms [31,32]. Scant literature focused on other possible biochemical correlates, such as brain-derived neurotrophic factor (BDNF), glutamate, N-acetylaspartate [33,34,35], or hormones [36]. However, a better understanding of biochemical correlates of both pathological and non-pathological video game use may help to shed light on risks and benefits of video games, which in the last few decades progressively became a widespread recreational activity and also a recognized sport field [37]. The present work aims to summarize and review the available literature about biochemical changes linked to video game use in IGD patients as well as in non-pathological users, and the eventual differences in between. This work may provide possible directions for future research on IGD treatment and, on the other hand, on the potential role of video games in therapeutic or preventive protocols for specific conditions.

## 2. Biochemical Correlates of IGD

### 2.1. Catecholamines

Several studies reported evidence of a dopaminergic pathway alteration among individuals with SUD [38] or behavioural addictions, such as morbid overeating [39,40] and pathological gambling (PG) [41]. According to the hypothesis that IGD may share similar neurobiological and biochemical alterations with SUD, many researchers have hypothesized dopaminergic system impairments in people with IGD. The first study, led by Kim et al. [42], highlighted a significant reduction of D2 receptors availability in the bilateral caudate and left putamen among IGD subjects (N = 5) when compared with seven age-matched healthy controls (HC), together with an inverse correlation between the severity of the addiction and the degree of DA receptor availability. A further research study using a PET scan with 11C-N-methylspiperone observed a deregulation of D2 receptors availability in the right inferior temporal gyrus during the resting state and a significant decrease in the putamen after a gaming task performed by 12 IGD patients and 14 HC [43]. Other studies focused on DA Transporter (DAT) levels. Hou et al., investigating the changes in the DAT striatal levels [44], found a significant decrease in the striatum among five subjects with IGD when compared with nine HC. Aritama et al. [45] examined the relationship between IGD, depressive syndrome, and DAT levels in 48 subjects assessed with the Internet Gaming Disorder Scale-Short form (IGDS9-SF), highlighting the presence of a lower DAT concentration and of an opposite correlation between IGD score and DAT levels. These authors stressed also how online games were reported to be associated with depressive symptoms in previous studies [46], while altered DAT levels were found in many disorders such as depression and bipolar disorder [47]. Liu and Lou [48] found, in a sample of 33 adolescents, significantly higher peripheral DA levels when compared with HC as well as positive correlations between plasma DA levels, the Internet Addiction Test (IAT) score, and the weekly online time. Conversely, Zhang et al. [49] observed a lack of significant difference in the levels of DA and serotonin (5-HT) between IGD subjects (20) and HC (15), but a lower level of norepinephrine (NE) in the IGD group. Another study reported significantly lower epinephrine (Epi) and NE levels among 118 subjects with IGD compared with 112 HC [50]. The IGD group reported also higher plasma levels of DA, although without reaching a statistically significant difference with respect to the HC group [50]. The authors hypothesized that the persistent stress induced by video gaming sessions may result in a reduction of Epi and NE plasma levels due to a receptor down-regulation, reflecting an adaptive response. Paik et al. integrated the evaluation of DA plasma levels with those of glutamate [35], on the basis of the evidence of a correlation between altered glutamatergic neurotransmission and several psychiatric disorders, including addictive disorders [51]. In particular, glutamate was hypothesized to be involved in pathophysiological mechanisms of PG [52]. The study highlighted lower glutamate concentrations in IGD patients (N = 26) than in HC (N = 23), with a lack of correlation between glutamate and DA serum levels and the time spent on gaming.

### 2.2. Hormones

The interest in hormonal changes among subjects with IGD is based on the growing evidence of the involvement of the hypothalamic–pituitary–adrenal (HPA) axis in both SUD and behavioural addictions [53]. An altered cortisol reaction to stress has been reported in PG, several behavioural addictions, and also in bulimia [54,55,56], leading authors to hypothesize similar alterations in IGD [53]. The first study in this field was Korean research [57] that evaluated the HPA axis activity and reported a higher level of adrenocorticotropic hormone (ACTH) and cortisol in the IGD group (N = 78) compared to the HC (N = 62). Geisel et al., in 2014, initially focused on cortisol, ACTH, and copeptin plasma levels, finding no differences between a group of IGD patients (N = 11), a group of 14 PG, and HC (N = 23) [58]. A study carried by Bibbey at al. [59], in a sample composed by 17 subjects with IGD, 28 subjects with alcohol use disorder (AUD), 17 subjects with comorbid IGD and AUD, and 26 HC, did not find an association between weakened cortisol stress response and IGD or AUD, either individually or in combination. On the other hand, Kaess et al. reported a more attenuated cortisol response in IGD patients (N = 24) than in HC (N = 25) [60]. Geisel et al., in a further study, shifted the focus on leptin plasma levels [61], which were reported to interact with the HPA axis, eventually decreasing its responsiveness to stress [62]. Authors compared plasma levels among subjects with IGD (N = 11), subjects with PG (N = 14), subjects with AUD (N = 39), and HC (N = 12), but they did not find any significant alteration. Koenig et al. also included in their study the evaluation of testosterone, progesterone, dehydroepiandrosterone (DHEA), and corticosterone, finding no differences between IGD patients (N = 31) and HC (N = 31) [63]. The most recent research in this field was conducted by Choi et al. [64], in a sample of 33 IGD subjects and 40 HC. Authors reported no significant differences in cortisol levels, together with a higher expression of melatonin and orexin A in IGD [64]. Orexin A was suggested to be involved in driving the motivation and in reward circuits associated with drug addiction [65].

### 2.3. Other Markers

Park et al. [15] focused on evaluating glucose metabolism alteration in a sample composed by 11 IGD and 9 HC. They found a significant increase in resting glucose metabolism among IGD subjects in the right middle orbitofrontal gyrus, the left caudate nucleus of the striatum (which is supposed to be strongly implicated in addiction and reward processing [66,67]) and the right insula (which plays a pivotal role in conscious urges to abuse drugs [68]). On the other hand, IGD subjects showed a significant decrease in the bilateral postcentral gyrus, the left precentral gyrus, the right superior parietal lobule, the right superior occipital gyrus, and the left inferior occipital gyrus. Another study [43] highlighted a significant increase of glucose metabolism among IGD patients (N = 12) compared with HC (N = 14) in the right supplementary motor area, right middle cingulum, and left superior medial frontal cortex during the resting state. A significant decrease was instead reported in the bilateral superior temporal poles and right orbitofrontal gyrus. Prefrontal reduction of glucose metabolism in these subjects is in line with the hypothesis that IGD may be associated with prefrontal dysfunctions similar to those reported in drug addictions [69]. In the same study, after a gaming task, authors reported decreased glucose metabolism in the right rectus, the right orbital part of the inferior frontal cortex, and the left orbital part of the superior frontal cortex exclusively in the IGD group. The lower number of regions with increased glucose metabolism in the IGD group may suggest a higher focus on the gaming task in IGD than in HC [43].

Other studies focused on BDNF, which was hypothesized to be involved in the development and maintenance of addictive disorders [70]. Previous studies also reported higher BDNF levels in PG [71,72,73]. However, Geisel et al. [74] did not find significant differences with respect to BDNF levels between patients with Internet use disorder (IUD) (N = 11) and HC (N = 10). According to these results, Choi et al. [64] confirmed the lack of differences in the expression levels of BDNF between subjects with (N = 33) or without (N = 40) IGD. They also did not find a difference in the expression levels of the chemokine RANTES, soluble intercellular adhesion molecule-1 (sICAM-1), and neural cell adhesion molecule (NCAM), which were reported to be altered in SUD [75,76,77,78]. However, Internet gaming time was negatively correlated with BDNF levels and positively correlated with sICAM-1 [64]. Jeong et al. [79] focused instead on Glial cell-derived neurotrophic factor (GDNF) plasma levels, due to its role in the behavioural effects of abused drugs [80], finding lowered plasma levels in IGD patients (N = 19) when compared with 19 HC. A negative correlation was also reported between GDNF levels and IGD severity, in line with a previous study which showed lower GDNF in AUD patients [81].

A single study led by Han et al. [82] focused on N-acetyl aspartate (NAA) and Choline (Cho). The authors reported lower levels of NAA in the right frontal cortex and of Cho in the right temporal cortex in the IGD group (N = 73) when compared with HC (38), together with a negative correlation between NAA levels and IGD severity [82]. NAA levels in the prefrontal cortex were also negatively correlated with perseverative errors on the Wisconsin Card Sorting Test (WCST), while the levels of Cho negatively correlated with Beck Depresion Inventory (BDI) scores. The authors suggested that these results, similar to those reported in patients with attention deficit hyperactivity disorder (ADHD) or major depressive disorder (MDD), may suggest the presence of deficits in the function of prefrontal and temporal cortex in IGD [82].

A more recent study [83] conducted an exploratory investigation on possible patterns of metabolic biomarkers in this field, reporting lower levels of myo-inositol in IGD patients (N = 24) when compared with 28 normal Internet users. This data may suggest a possible link with IGD and MDD, a disorder frequently associated with reduced myo-inositol levels in the prefrontal cortex, possibly due to aberrant glial metabolism [84]. They also found, in the IGD group, altered valine-leucine-isoleucine and glycine-serine-threonine pathways and lower levels of arabitol and glyceric acid. IGD severity score was positively correlated with aspartate, pyrrole-2-carboxylic acid, cholic acid, uracil, and nicotinamide levels, but negatively correlated with sugar alcohols and common fatty acids levels [83].

Finally, Lee et al. [85] compared miRNA profiles between 25 subjects with IGD and 26 HC, following the evidence that miRNA expression profiles may be altered in brain tissue of patients with psychiatric disorders [86,87] and may eventually be considered as potential biomarkers for some of them [88,89]. Results showed that the expression of three miRNAs, which were commonly reported to be involved in many neuropsychiatric disorders, was significantly lower in IGD: hsa-miR-26b-5p and hsa-miR-652-3p (whose expression was altered in schizophrenia [90,91]), and hsa-miR-200c (which was found down-regulated in MMD [92]). On the other hand, it should be noted that, on the basis of these works, miRNA might have a good sensitivity, but as a potential biomarker, it seems to lack the sensitivity and thus the capability of specifically identifying subjects with IGD, especially considering that similar results were reported among patients with other psychiatric conditions [86,87,88,89,90,91].

### 2.4. Genetics

Han et al. [93] focused on DA genes and found that low activity Catechol-O-methyltransferase (COMT) alleles were more frequent among subjects with excessive Internet game play (EIGP) (N = 79) than in HC (75). The overall frequency of genotypes containing DA receptor D2 (DRD2) Taq1A1 was also higher among EIGP subjects, and it was positively correlated with reward-dependence levels as measured by the Reward-Dependence (RD) scale. This latter gene has been extensively studied in AUD [94] and PG [95], highlighting that a reduced DRD2 density in the striatum might increase the risk for addictive, impulsive, and compulsive behaviours [96]. Lee et al. [97] reported a higher prevalence of the homozygous short allelic variant of the serotonin transport gene (*SS-5HTTLPR*), which was associated in previous studies with AUD [98] and MDD [99], among subjects with excessive Internet use (EIU) (N = 91) when compared with HC (N = 75). Montag et al. [100] assessed the frequency of the T allele (CC genotype) of rs1044396 in the nicotinic acetylcholine receptor alpha 4 subunit (CHRNA4), which previous studies linked to AUD [101], PG [102], higher anxiety traits [103], and higher levels of depression [104]. The T-variant occurred significantly more frequently in the IGD group (N = 132) than in HC (N = 132). Similar results came from a later study by Jeong et al., conducted in a smaller sample of 30 IGD and 30 HC [105]. Park et al. [106] highlighted an increased frequency of the AA genotype of corticotropin releasing hormone receptor 1 (*CRHR1*) (rs28364027) gene in the IGD group (N = 118) compared to HC (N = 112), which have been previously associated with AUD [107]. More recently, Kim N. et al. [108] investigated leukocyte telomere length (LTL) in a sample of students with (N = 118) or without IGD (N = 112) due to its possible link to many psychiatric conditions [109]. In line with studies which highlighted a relationship between LTL and drug addiction [110], the results showed a significantly shorter LTL in IGD subjects and a positive correlation with LTL, Epi, and NE levels.

All the studies are summarized in Table 1.

## 3. Biochemical Correlates of Video Gaming among Non-Pathological Users

### 3.1. Cathecolamines

Due its well-known role in learning, sensorimotor integration, reinforcement of behaviour, and attention [111,112], DA has been the first catecholamine to also be investigated in non-pathological gaming settings. The first study in this field was led by Koepp et al. in 1998 [113], featuring a [11C] RAC–PET to assess DA release during a video gaming session in eight healthy subjects. Results showed a reduced binding potential during the video game, particularly in the ventral striatum, consistent with an increased release and a higher binding of DA to its receptors. Similar outcomes came from a later study by Weinstein et al. [114], where DA release was compared between nine former ecstasy users and eight HC after a motorbike gaming task. A significant reduction in binding potential was highlighted in the caudate after the performance compared with the baseline measurement, while there was no reduction among the former ecstasy users, probably due to previous sensitization to stimulant drugs. These results suggest that video game playing may cause a DA release similar to the effects of psycho-stimulant drugs such as methylphenidate [115,116] and amphetamine [117]. This DA alteration may eventually be involved in the development of addictive behaviours related to video games in vulnerable subjects [114].

NE and Epi were firstly investigated by Eisenhofer et al. in 1985 [118]. In this study, the authors compared Epi and NE levels in 25 healthy subjects during a competitive electronic game or a cognitive task. A significant increase of Epi plasma levels during the competitive electronic game was highlighted, similar to the one obtained during the cognitive task. Although no difference was found in NE plasma levels, it should be noted that the alteration of NE plasma levels in response to stress is age dependent [119], and the young age of the subjects recruited for the study may have influenced this result. NE was also investigated by Skosnik et al. [120] through dosing salivary alpha-amylase (sAA), whose increase was associated in previous literature to NE increase [121]. The study highlighted higher sAA levels among 20 healthy subjects after a violent video game, indicating a possible increase of NE as well [121]. Ten years later, Maass et al. [122] compared the effect of violent elements of video games on sAA changes in a sample of 88 healthy subjects. Subjects were divided in four groups based on the activity (violent video game, non-violent video game, violent TV show, non-violent TV show). The non-violent video game group showed significant lower levels of sAA when compared with the violent video game group. A further study examined Epi and NE in 12 children with type 1 diabetes during a video gaming session and a reading session [123] and found a higher increase in both Epi and NE levels during the video game session (although only the NE increase was statistically relevant), suggesting that video game playing may induce enough stress to stimulate the peripheral sympathetic system.

### 3.2. Hormones

Research concerning hormone secretion during video gaming has mainly focused on cortisol levels, starting with a first study in 1998 [18] that measured cortisol levels in 10 healthy subjects before and after a gaming session: authors reported decreased cortisol levels after the task. Similar results came from a later study [124] where cortisol levels were evaluated among 43 healthy subjects before and after a gaming task, highlighting a decrease in the latter situation. Alivary et al. [125] also reported a significant decrease in cortisol levels after playing a soccer video game. However, other authors did not find a significant difference in cortisol levels related to video gaming among non-pathological users. Phan Hug et al. [123] reported no changes in both cortisol and growing hormone (GH) levels among 12 children with type 1 diabetes after the gaming task, although they hypothesized that this result may be explained by the delayed response of the HPA. On the other hand, Yui et al. [126] evaluated the effects of video game play on plasma cortisol in 10 patients with autism spectrum disorder (ASD) and seven HC under a stressful situation, commonly able to increase cortisol levels, such as an interview from unfamiliar adults. Interviews were preceded and followed by video gaming sessions. In both groups, no significant differences were found between plasma cortisol levels at 28 days before the interviews and at 5 min after the interviews, suggesting that video game play may attenuate plasma cortisol response to a stressful setting, possibly acting as a distracting factor [126]. More recently, in 2017, Gray et al. [36] found no difference in cortisol levels among 26 healthy subjects playing a competitive video game (*League of Legends*) against other people or against the computer.

Following the spreading hypothesis of a supposed link between media violence (such as movies [127], video games [128,129], and song’s lyrics [130]) and aggressive behaviors, many researchers focused on the possible influence that violent elements in video games may exert on cortisol response. Skosnik et al. [120] analyzed salivary cortisol levels after playing a violent video game but found no difference compared to the baseline. More recently, Hebert et al. hypothesized that music might play a crucial role in the stress generated by video game playing [131]. They examined the effect of built-in music on cortisol secretion comparing 26 healthy subjects playing in silence and 26 healthy subjects playing with techno background music, and reporting significantly higher cortisol levels in the music group. This result was in line with a previous study that examined the physiological effects of this kind of music [132]. Ivarsson et al., in 2009 [133], compared salivary cortisol levels in 21 boys after playing a violent video game and after playing a non-violent video game: results showed a significant fall in the cortisol levels after playing without differences with respect to the type of game. One year later, Maass et al. [122] reported, among 96 healthy subjects, a significant decrease of cortisol levels with respect to the baseline after a video gaming session without significant differences between violent and non-violent video games. Conversely, one recent study [134] analyzed the changes in cortisol levels in 136 healthy subjects randomly assigned to play a violent or a non-violent video game, finding significantly higher cortisol levels during game play in the violent game group. These results suggest that violent video game play may activate the sympathetic nervous system and engage the fight-or-flight response, resulting in increased stress hormone release.

Other studies focused on testosterone, with interesting results. Oxford et al. [135] evaluated salivary testosterone in 14 teams, each made of three male players, after competing in within-group and between-group violent video games. Results showed a significant increase in testosterone only when the between-group tournament was played before the within-group tournament. These findings may suggest that contributing to an in-group team’s victory while playing a video game may elicit the same testosterone responses that are triggered during men’s physical coalitional competition [136]. The above-mentioned study by Gray et al. [36] also investigated changes in testosterone and amplified their search field to DHEA, androstenedione, and aldosterone. Hormone levels were assessed in 26 healthy subjects while playing a competitive video game (*League of Legends*) against other people or against the computer. Results showed no changes in testosterone, DHEA, or androstenedione levels in either of the settings, while aldosterone levels were significantly reduced in both. When playing against other people, a positive correlation was found between the duration of the game and the increase in testosterone, DHEA, and androstenedione. In this framework, the lack of a significant increase in the hormone’s levels might also be correlated to the short duration of the game (15–27 min), in line with a previous study that suggested how increases in testosterone are more likely to occur in competitions longer than 15 min compared to shorter ones [137].

### 3.3. Other Markers

The interest in energy expenditure and glucose levels while and/or after video game playing rose after the reported evidence about a correlation between the number of hours spent daily playing electronic games and an increased risk of obesity [138,139,140]. In this framework, Segal and Dietz aimed to quantify the energy cost of video game playing [19] and reported that in a sample of 32 healthy subjects, video games increased the energy expenditure by roughly 80% (similarly to walking at a pace of 2.0 mph [141]). In 2006, Wang and Perry examined the metabolic changes that occur while playing a video game among 21 healthy subjects [142], focusing on glucose and lactate concentrations. They reported an increase in lactate compared with the baseline, although not reaching a statistical relevance. Glucose showed instead a non-significant decrease, in contrast to what has been observed in response to a physical exercise. [143] Later Phan-Hug et al. [123] investigated the possible influence of video games on glycemic balance in children with type 1 diabetes (N = 12), reporting higher glycemic values during a video gaming session than during resting periods, although no significant difference was found between video gaming and reading sessions. A further study [43] investigated glucose metabolism in 14 healthy males and 12 IGD patients (results about the differences between groups are described in the previous chapter). Authors reported also that, in the within-group analysis, healthy subjects showed a significant increase in the glucose metabolism in the right cuneus and right calcarine and a decrease in the left medial temporal cortex and left medial frontal cortex after the gaming task. On the other hand, in the IGD group was found an increased metabolism in the right lingual gyrus, left cerebellum, and calcarine, together with a decrease in the left medial part of the superior frontal cortex, left rectus, and left superior frontal cortex in the IGD group. Both groups reported increased glucose metabolism in the occipital region, and a decreased amount in the prefrontal and temporal regions after the gaming task. On the basis of this result, authors hypothesized that video games may modify visual selective attention [144] and cortical networks for complex visual motor transformations [145], considering the role of the occipital region as the visual center and its close association with vision stimulation. Finally, Chaput et al. [146] reported significantly higher energy expenditure and significantly increased plasma glucose concentrations during the video game play condition compared with the resting state in 22 healthy subjects, but no difference in cortisol, ghrelin, and insulin levels.

All the studies are summarized in Table 2.

## 4. Discussion

Since the spreading of video game use in the general population, most of the studies in the field of brain sciences have been inclined to focus on possible harmful consequences of video gaming from both a medical and psychosocial point of view [147,148], such as the development of behavioural addiction [149,150] or the increase in aggression [148]. As reviewed above, studies about IGD were mostly focused on DA and often reported how this condition seems to show biochemical correlates similar to those reported in behavioural addictions and SUD, suggesting the presence of shared pathological mechanisms [35]. In particular, IGD subjects showed reduced levels of DA D2 receptor availability in the striatum [42] and reduced striatal DA transporter (DAT) availability [44]. Moreover, male individuals affected by IGD reported a relevant decrease in glucose metabolism in the prefrontal, temporal, and limbic regions, together with lower levels of D2 receptor availability in the striatum [43]. The D2 receptor-mediated dysregulation of the orbitofrontal cortex (OFC) may also underlie the loss of control and compulsive behaviours observed in IGD. The increased activity of the DA reward system, together with the lower D2 receptor occupancy and the reduced DAT density, seems to be similar to the down-regulation reported among subjects with SUD, supporting a behavioural addiction model of IGD [35]. Such a perspective would also be reinforced by the evidence that DA genes (Taq1A1 variation of DA D2 receptor and low activity Val158Met in the catecholamine-O-methyltransferase alleles) [93] and serotoninergic genes (SS-5HTTLPR), interacting with personality and environmental factors, may play a role in shaping the vulnerability towards IGD [97], eventually leading to a conceptualization of IGD as a reward deficiency syndrome [35,96]. A lower number of studies focused on different markers, reporting controversial results. Some researches seem to confirm the above-described model, highlighting, among subjects with IGD, lower serum levels of glutamate [34], or lower levels of NAA in the right cortex and of Cho in the medial temporal cortex [35]. Despite that, studies on cortisol and BDNF often did not find significant differences between IGD patients and HC [64,73,74], suggesting the possible presence of specific characteristics in this condition with respect to other addictive disorders [70,73]. However, it should be noted that literature about this topic is still very limited, especially for biochemical correlates other than DA, and further studies are needed to clarify the neurobiological underpinnings of IGD.

Studies about biochemical changes related to video gaming among non-pathological users, on the other hand, showed a more complex picture. Studies on DA seem to confirm the association between gaming and DA release, stressing the addictive potential of this activity [113,114], while other research seems to show that video gaming may have alternatively a beneficial or negative influence on stress mechanisms. In particular, some studies found that video gaming sessions may enhance NE and sAA levels [120,123], suggesting that video games may be able to stimulate the peripheral sympathetic system. Despite that, most of the studies on HPA axis in this field reported reduced levels of cortisol after the gaming task, stressing also a possible role of video games in attenuating stress response, acting as a distracting factor in stressful settings [126]. This data is also in line with the reported beneficial use of video games as a cognitive distraction in order to modulate pain perception [151,152,153], in particular during chemotherapy cycles for children [151,154,155] or among subjects with sickle cell anemia [156]. Research on eventual differences between violent and non-violent video games led to controversial results, alternatively showing enhanced cortisol levels associated to violent games [134] or reduced cortisol levels after gaming sessions with both violent or non-violent games [133]. These latter findings are in line with recent evidences about the lack of association between violent video games and aggressive behaviours [157]. Studies on glucose metabolism highlighted that video gaming was related to a significant energy expenditure, and to an enhanced glucose metabolism in specific brain areas, leading to authors hypothesizing, according to other neuroimaging studies, a potential role of video gaming in shaping visual selective attention and visual motor transformation [43,144,145,146]. As hypothesized by other authors, it is possible that the specific kind of video games (e.g., casual vs. competitive), and the specific individual approach to them, may lead to different, even opposite modulations of the stress response and possibly of other neurobiological outcomes. Globally, biochemical studies in this field are still very limited, and further research is needed to clarify how the specific kind of video game, and, eventually, personal factors, may influence the outcome in terms of neurobiological changes.

### Further Directions and Therapeutic Perspectives

In the last few decades, several authors focused on evaluating the possible use of video games for increasing well-being and promoting individual growth [158]. Video games use a multimedia language, based on fluid models of intervention and, in addition to being a source of entertainment, they may be also considered a versatile tool with high educational potential [158], which may also help to improve language difficulties [159], problem solving strategies [160], and mathematical skills [161]. The inclusion of video games among the intervention strategies among children with learning disabilities was hypothesized several years ago [162,163]. In the video game dimension, there are various spaces of representation, rules, and ways of learning skills that involve the gamer, making him or her feel as an active part of the process [164]. Video gaming is considered a fun and stimulating activity, that may also enhance the maintenance of both individual and shared attention [165]: this is in line with the above-reported studies, which highlighted changes in DA release and glucose metabolism in specific brain areas in association with video game use [43,114]. Some authors stressed also that the video game space may share some features with the *“joint attentional scene”*, such as the cognitive stimulation towards understanding the intentionality of the other participants, the specific organization of communication methods and behavioural synchronization, the ability of changing perspective and point of view, the possibility of learning new skills and the perception of one-self as a subject within a specific community [166]. In this framework, video games were reported to exert potential beneficial effects in the context of cognitive rehabilitation and psychotherapy, especially among children and adolescents, who are more accustomed to using these technologies and may feel more motivated and stimulated by the video game setting towards reaching the therapeutic objectives [167,168].

Several authors reported cognitive improvements related to video game use in healthy individuals, such as positive changes in reaction times, implementation of conceptual thinking, executive functioning, attention, memory, creativity, improvement of hand eye coordination, and spatial visualization ability [144,169,170,171,172]. A meta-analysis conducted some years ago reported also that action/violent and mimetic games seem to show larger effects than non-action and puzzle games on the improvement of information processing [172]. According to these findings, video games were reported to be a useful tool in order to equalize individual differences in spatial skill performance among subjects with poor basic skills, or in order to gain cognitive improvements among older adults [169,171,173,174]. Moreover, other research stressed the utility of video gaming for cognitive and muscular training in patients with brain damage or physical injuries, neurodegenerative diseases, and muscular dystrophies [175,176,177,178,179]. In the field of mental health, several studies reported beneficial effects of specifically designed video games (which often include elements of cognitive behavioural therapy) in generalized anxiety disorders and specific phobias [180,181,182,183,184,185,186], in eating disorders [187], and in ASD [186,188]. In this latter group, improvements were reported in stereotyped movements, social skills, and cognitive functions [186,189]. Among subjects with ADHD, video game play seems to inhibit hyperactivity and increase levels of attention [186,190]. Other researchers reported that among patients with depression, playing commercial action video games improved executive function and reduced rumination [191]. Neuroimaging studies highlighted possible neurobiological correlates of the above-reported cognitive improvements related to video game use: e.g., an increase of gray matter was highlighted in several brain areas, such as ventral striatum, dorsolateral prefrontal cortex, hippocampus, and bilateral cerebellum in association with video game training [192,193]. Noticeably, these neuroplastic effects suggest that video games might exert a beneficial effect in disorders which feature a volume reduction of the hippocampus or the prefrontal cortex, such as post-traumatic stress disorder, schizophrenia, and dementia [194]. Grey matter increases in adults seem to also be positively related to the amount of lifetime video gaming [193], while changes in the enthorinal cortex seem to be positively predicted by the use of platforms or logic/puzzle games and negatively by action and roleplay ones [193]. In another study, the increase of gray matter was positively correlated with the use of allocentric strategies and the desire to play [194]. Some authors hypothesized a link between this data and the increased DA release during video game play reported by previous studies [113], stressing the crucial role of DA in both hippocampal and prefrontal cortex plasticity as well as its central role in reward mechanisms [193]. Despite that, no study specifically evaluated the biochemical correlates of video gaming when used as a therapeutic strategy or as a tool for cognitive improvement. Further studies should address this topic in order to shed light on the possible applications of video games as a form of therapeutic or training strategy, as well as on possible related risks, including the addictive potential. As reported above, results about biochemical correlates of video games in non-pathological users, such as the modulation of DA and cortisol levels, may allow formulating hypotheses about neurobiological underpinnings of the possible beneficial effects related to video games, eventually providing useful indications for further research in this field. The main limitation of the present study lies in its methodology. Since biochemical research on video game use in different populations is a very novel field, although bearing wide implications, actual research is scant and discrete (a point that has been also underlined before). Given the current state of the literature, we found it more informative to approach the available research data with a narrative methodology, albeit weaker from an epistemic point of view, in order to maximize the dissemination of knowledge on this subject. Further research with standardized approaches is needed in order to better highlight the implications of findings in this field.

## 5. Conclusions

Globally, results on biochemical correlates of video gaming seem to suggest interesting perspectives, highlighting both potentially harmful (such as addictive potential and altered stress responses) and potentially beneficial (such as stress control or cognitive improvements) consequences. In this framework, the activity of video gaming should be considered a variable whose effects may vary depending on the specific kind of game, on environmental contexts, and on individual vulnerabilities. From a clinical point of view, it is of the utmost importance to further investigate the specific mechanisms that may lead to different pathways. Further studies should aim to improve our knowledge on this subject, in order to clarify when video game use should be recommended, limited, or avoided, depending on the need of the individual.

## Figures and Tables

**Table 1:** Studies about biochemical correlates of IGD.

Reference	Participants	Main Findings
[42] Kim S. H. et al. (2011)	IAD patients N = 9 (M); Mean age = 22.60 ± 1.16. HC N = 7 (M); Mean age = 23.14 ± 0.67. Assessed with IAT and IADDC.	A significant reduction of DA D2 receptors availability in the bilateral caudate and left putamen along with an inverse correlation between the severity of the addiction and the degree of dopamine receptor availability. *Method: PET scan with [11C] raclopride*
[44] Hou H. et al. (2012)	IAD patients N = 5 (M); mean age = 20.40 ± 2.30. HC N = 9 (M); mean age = 20.44 ± 1.13 years. Assessed with IADQ and IADDC.	A significant decrease of DAT expression level of striatum. *Method: SPECT with 99mTc-TRODAT-1*
[43] Tian M. et al. (2014)	IGD patients N = 12 (M); mean age = 23.50 ± 2.58. HC N = 14 (M); mean age = 22.71 ± 1.27. Assessed with IAT.	Lower-11C-NMSP binding levels in the right inferior temporal gyrus in the resting state and, after the Internet gaming task, lower binding levels in the putamen. IGD group: A significant decrease in glucose metabolism in the bilateral superior temporal poles and right orbitofrontal gyrus, but a significant increase in the right supplementary motor area, right middle cingulum, and left superior medial frontal cortex, in the resting state. After the Internet gaming task, a significant decrease in the metabolism in the right rectus, the right orbital part of the inferior frontal cortex, and the left orbital part of the superior frontal cortex. *Method: PET and 11C-N-methylspiperone and 18F-fluoro-D-glucose*
[45] Ariatama B. et al. (2019)	IGD patients N = 48; F = 2; M = 46. Assessed with IGDS9-SF.	IGD group: Lower DAT concentration and a strong opposite correlation between IGD and DAT levels.
[48] Liu M. & Lou J. (2015)	IAD patients N = 33; F = 5; M = 28; mean age = 16.58 ± 2.29. HC N = 33; F = 5; M = 28; mean age = 16.58 ± 2.29. Assessed with IAT.	IGD group: A significantly higher DA plasma level, positively correlated with the IAT score and the weekly online time.
[49] Zhang H-X. et al. (2013)	IGD patients N = 20; F = 2; M = 18; mean age = 16.86 ± 1.8. HC N = 15; F = 2; M = 13; mean age = 18.16 ± 2.7. Assessed with DQIA.	IGD group: A lower mean level of NE but no significant difference in the levels of 5-HT and DA.
[50] Kim N. et al. (2016)	IGD patients N = 118 (M); mean age = 16.64 ± 1.13. HC N = 112 (M); mean age = 16.63 ± 0.89. Assessed with OGASA.	IGD group: Significantly lower plasma levels of Epi and NE, and not significantly higher levels of DA. Slight negative correlation between daily Internet gaming time and plasma NE levels.
[34] Paik S-H. et al. (2018)	IGD patients N = 26 (M); mean age = 28.16 ± 5.235. HC N = 23 (M); mean age = 28.16 ± 5.235. Diagnosed according to DMS-5.	IGD group: Lower glutamate concentrations and no difference in DA serum levels.
[57] Kim E. H. & Kim N. H. (2013)	IGD patients N = 78 (M). HC N = 62 (M).	Significantly higher serum cortisol and ACTH levels.
[58] Geisel O. et al. (2015)	IGD patients N = 11(M); mean age = 27.5 ± 5.2. HC N = 10 (M); mean age = 30.5 ± 5.6. PG N = 14 (M); mean age = 35.4 ± 9.5. HC N = 13(M); mean age = 35.2 ± 8.4 Diagnosed according to DSM-5 and ICD-10.	No differences were found in the cortisol, ACTH and copeptin plasma levels.
[59] Bibbey A. et al. (2015)	IGD patients N = 17; F = 12; M = 5; mean age = 19.8 ± 1.29. AUD patients N = 28, F = 15; M = 13; mean age = 19.2 ± 0.67. Comorbid patients N = 17; F = 13; M = 4; mean age = 22.3 ± 2.32. HC N = 26; F = 28; M = 8; mean age = 20.5 ± 2.94. Assessed with IAT and PIUS.	Neither problematic Internet behaviour nor excessive alcohol consumption, either individually or in combination, were associated with blunted cardiovascular or cortisol stress reactions.
[60] Kaess M. et al. (2017)	IGD patients N = 24 (M); mean age = 18.38 ± 3.39 HC N = 25 (M); mean age = 19.68 ± 3.4. Assessed with CSV-S.	Attenuated cortisol response in IGD during a standardized laboratory stress task.
[61] Geisel O. et al. (2018)	IGD patients N = 11 (M); mean age = 27.5 ± 5.1. PG patients N = 14 (M); mean age = 35.4 ± 9.5. AUD patients N = 39 (M); mean age = 45.9 ± 7.3. HC N = 12 (M); mean age = 36.6 ± 8.3. Diagnosed according to DSM-5 and ICD-1.	Leptin plasma levels of the patients did not differ between the groups. In patients with IGD, leptin plasma levels were positively correlated with BDNF serum levels.
[63] Koenig J. et al. (2019)	IGD patients N = 31 (M); mean age = 18.77 ± 3.01. HC N = 31(M); mean age = 20.06 ± 2.94. Diagnosed according to DSM-5.	No evidence for altered hair hormones, including cortisol, cortisone, testosterone, progesterone, DHEA, and corticosterone, was found in patients with IGD *Method: liquid chromatography-mass spectrometry*
[64] Choi M. R. et al. (2020)	IGD patients N = 33; F = 7; M = 26; mean age = 15.18 ± 0.31. HC N = 40; F = 10; M = 30; mean age = 15.08 ± 0.24. Diagnosed according to DSM-5.	The expression levels of orexin A and melatonin were significantly elevated in IGD; oxytocin levels tended to be higher but the difference was not statistically significant. The expression levels of cortisol did not differ between the groups. The expression levels of BDNF, sICAM-1, RANTES, and NCAM did not differ between the groups. Internet gaming time was significantly negatively correlated with BNDF expression levels and positively correlated with sICAM-1 expression levels. *Method: Human Neuropeptide Magnetic Bead Panel, Human Circadian/Stress Magnetic Bead Panel, Human Neurodegenerative Disease Magnetic Bead Panel 3*
[15] Park H. S. et al. (2010)	IGD patients N = 11 (M); mean age = 23.5 ± 2.9. HC N = 9 (M); mean age = 24.7 ± 2.4. Assessed with IGS.	A significant increase in resting glucose metabolism in the right middle orbitofrontal gyrus, the left caudate nucleus, and the right insula and a significant decrease in glucose metabolism in the bilateral postcentral gyrus, the left precentral gyrus, and the right superior parietal lobule, as well as in the right superior occipital gyrus and the left inferior occipital gyrus. *Method: 18F-fluorodeoxyglucose PET scan*
[74] Geisel O. et al. (2013)	IUD patients N = 11 (M); mean age = 27.57 ± 5.1. HC N = 10 (M); mean age = 30.57 ± 5.6. Diagnosed according to DSM-5.	No difference in BDNF serum levels between the two groups.
[79] Jeong J-E. et al. (2019)	IGD patients N = 19 (M); mean age = 31.2 ± 8.0. HC N = 19 (M); mean age = 31.3 ± 4.2. Assessed with IAT.	IGD group: Lower GDNF plasma levels; negative correlation between GDNF levels and IAT score.
[82] Han D. H. et al. (2014)	PGA patients N = 73; F = 5; M = 68; mean age = 20.6 ± 5.1. HC N = 38; F = 3; M = 35; mean age = 22.0 ± 5.2. Assessed with YIAS, BDI, WSCT.	IGD group: Decreased levels of NAA in right frontal cortex and decreased levels of Cho in the right temporal cortex. Negative correlation between NAA levels and IAS, WSCT scores. Negative correlation between levels of Cho in the right temporal cortex and BDI score. *Method: structural MR and 1H-MRS*
[83] Cho Y. U. et al. (2017)	IGD patients N = 24 (M); mean age = 23.7 ± 2.3. HC N = 28 (M); mean age = 23.7 ± 2.3. Assessed with IAT.	IGD group: Decreased levels of myo-inositol, arabitol, and glyceric acid. A positive correlation between Internet addiction severity score and aspartate, pyrrole-2-carboxylic acid, cholic acid, uracil, and nicotinamide levels and a negative correlation one with sugar alcohols and common fatty acids. Altered valine-leucine-isoleucine and glycine-serine-threonine pathways.
[85] Lee M. et al. (2018)	IGD patients N = 25. HC N = 26. Diagnosed according to DSM-5.	IGD group: Significantly lower expression of three miRNAs (hsa-miR-200c-3p, hsa-miR-26b-5p, and hsa-miR-652-3p) reported to be involved in many neuropsychiatric disorders. *Method: TaqMan Low Density miRNAArray*
[93] Han D. H. et al. (2007)	EIGP subjects N = 79 (M); mean age = 16.08 ± 0.66. HC N = 75 (M); mean age = 16.07 ± 0.68. Assessed with IAS, RD	EIGP group: The DRD2 Taq1A1/A1 genotype was more frequent and the frequency of genotypes containing DRD2 Taq1A1 was higher. COMT genotype did not differ, but the frequency of genotypes containing COMT^L^ was higher. Positive correlation between RD score and the presence of DRD2 Taq1A1.
[97] Lee Y. S. et al. (2008)	EIU patients N = 91 (M); mean age = 16.1 ± 0.7. HC N = 75 (M); mean age = 16.1 ± 0.7. Assessed with IAS.	EIU group: Higher prevalence of the homozygous short allelic variant of the serotonin transporter gene (*SS-5HTTLPR*).
[100] Montag C. et al. (2012)	IGD patients N = 132; F = 52; M = 80; mean age = 23.92 ± 5.23. HC N = 132; F = 52; M = 80; mean age = 23.92 ± 5.23. Assessed with IAT.	IGD group: The T-variant occurred significantly more frequently.
[105] Jeong J-E. et al. (2017)	IGD patients N = 30 (M); mean age = 30.73 ± 6.72. HC N = 30 (M); mean age = 30.57 ± 5.48). Assessed with IAT.	IGD group: Lower numbers of the T allele of rs1044396 in the nicotinic acetylcholine receptor alpha 4 subunit (*CHRNA4*).
[106] Park J. et al. (2018)	IGD patients N = 118 (M); mean age = 16.63. HC N = 112 (M); mean age = 16.63. Assessed with KADO scale.	No difference in the DRD4 and DAT1 VNTR polymorphisms and in the *NET8* (rs5569) or *CHRNA4* (rs1044396) genes. Increased frequency of the AA genotype of *CRHR1* (rs28364027) in the IGD group.
[108] Kim N. et al. (2019)	IGD patients N = 118 (M); mean age = 16.64 ± 1.13. HC N = 112 (M); mean age = 16.63 ± 0.89. Assessed with OGASA.	IGD group: Leukocyte telomere length is significantly shorter and is positively correlated to Epi and NE levels.

Beck Depressive Inventory: BDI; Internet Addiction Test: IAT; Internet Addictive Disorder Diagnostic Criteria: IADDC; Internet Addiction Diagnostic Questionnaire: IADQ; Diagnostic Questionnaire for Internet Addiction: DQIA; Online Game Addiction Scale for Adolescents: OGASA; Pathological Internet use scale: PIUS; Scale for the Assessment of Pathological Computer-Gaming: CVS-S; Korean Internet Game Addiction Scale: IGS; IAS: Internet Addiction Scale; RD: Reward-Dependence scale; Wisconsin Card Sorting Test: WCST; Young Internet Addiction Scale (YIAS).

**Table 2:** Studies about biochemical correlates of non-pathological video gaming.

Reference	Participants	Main Findings
[113] Koepp M. J. et al. (1998)	Healty subjects N = 8 (M); range 36–46 years	Reduced DA binding potential during the video game (VG), particularly in the ventral striatum. Methods: [11C] RAC–PET
[114] Weinstein A. M. (2010)	Former ecstasy users N = 9; F = 1; M = 8; mean age = 25 ± 3.5HC N = 8; F = 1; M = 7; mean age = 35.75 ± 6.5	Significant reduction in DA binding potential in the caudate after the performance, compared to the baseline, exclusively among HC. *Game: motorbike-riding computerized video game by “motoGp”* Methods: [123I] IBZM SPECT
[118] Eisenhofer G. et al. (1985)	Healthy subjects N = 25 (M); mean age 24.4	Significant increase in Epi plasma levels during the VG task but no significant changes in NE plasma levels.
[120] Skosnik P. D. et al. (2000)	Healthy subjects N = 20; F = 10; M = 10; mean age = 21.3	Increase in the a-amylase salivary levels after a violent VG, but no change in the cortisol salivary levels.
[122] Maas A. et al. (2010)	Healthy subjects N = 98 (M); mean age = 12.77 ± 0.74	Significant lower levels of sAA in the non-violent VG compared to the violent VG group. Significant decrease from the baseline cortisol levels in the non-violent VG and in the violent VG. *Games: “Who wants to be a millionaire? Junior Edition”; “King Kong”*
[123] Phan-Hug F. et al. (2011)	Diabetes 1 patients N = 12; F = 5; M = 7; mean age = 10.5 ± 1.3	Significant increase of NE during VG session, non-significant increase of E. No differences in cortisol and GH levels. Trend towards higher glycemic values during the VG session, a significant difference in the glycemic values between the VG resting period and the reading resting period. *Game: “Harry Potter and the Chamber of Secrets”*
[18] Denot-Ledunois S. et al. (1998)	Healthy subjects N = 10; F = 6; M = 4; mean age 9.2 ± 1.5	Decreased cortisol levels. *Game: “Tetris”*
[124] Sharma R. et al. (2006)	Healthy subjects N = 43; F = 4, M = 39; range 18–30 years	Decreased cortisol levels after the gaming session. *Games: “Tetris”; “Blackhawk Striker Launch”; “Starship Eleven”*
[125] Aliyari H. et al. (2015)	HC N = 32; mean age = 20	Significant decrease in cortisol levels after the game. *Game: “Fifa 2005”*
[126] Yui K. et Ohnishi M. (2013)	ASD patients N = 10; F = 2; M = 8; mean age = 10.90 ± 4.04 HC N = 7; F = 4; M = 4; mean age = 11.71 ± 4.11	No differences in plasma cortisol levels between the groups at 28 days before the interviews, and at 5 min after the interviews. No difference in plasma cortisol levels between 28 days before and 5 min after of the in interviews in the ASD patients, and also in the HC.
[36] Gray P. B. et al. (2018)	Healthy subjects N = 26 (M); mean age = 20.46 ± 1.42	No significant changes in testosterone, cortisol, DHEA, and androstenedione levels while playing against a computer or another person. A significant decrease in aldosterone levels in both setting. A positive correlation between the duration of the game and the increase in testosterone, DHEA, and androstenedione while playing against another person.
[131] Hebert S. et al. (2005)	Healthy subjects N = 52 (M); mean age = 24.3 ± 3.1	Subjects playing with techno background music (N = 26) showed significantly higher cortisol levels after the gaming task.
[133] Ivarsson M. et al. (2009)	Healthy subjects N = 21 (M); mean age = 13.3 ± 0.7	Significant fall in the cortisol levels after playing, but no difference between the two playing conditions (violent and non-violent). *Game: “Manhunt”; “Animanics”*
[134] Gentile D. A. et al. (2017)	Healthy subjects N = 136; F = 67; M = 67; unmarked = 2; mean age = 10.1 ± 1.25	Significantly higher cortisol levels during game play in the violent game group. *Games: “Spiderman”; “Finding Nemo”*
[135] Oxford J. et al. (2010)	Healhty subjects N = 42 (M); mean age = 19 ± 0.97	A significant increase in testosterone when the between-group tournament was played before the within-group tournament. Game: “Unreal Tournament 2004”
[19] Segal K. R. et al. (1991)	Healthy subjects N = 32; F = 12; M = 20; mean age = 20 ± 1	Increased energy expenditure by roughly 80%. *Game: “Ms Pac-Man”*
[142] Wang X. et al. (2006)	Healthy subjects N = 21 (M); mean age = 8.8 ± 1.1	Lactate levels increased from the baseline without reaching a statistical relevance. Glucose showed a non-significant decrease. *Game: “Tekken 3”*
[43] Tian M. et al. (2014)	IGD patients N = 12 (M); mean age = 23.50 ± 2.58 HC N = 14 (M); mean age = 22.71 ± 1.27	Increased glucose metabolism in the right cuneus and right calcarine; decreased in the left medial and temporal cortex and left medial frontal cortex after the gaming task in HC. Increased metabolism in the right lingual gyrus, left cerebellum, and calcarine, alongside a decrease in the left medial part of the superior frontal cortex, left rectus, and left superior frontal cortex in the IGD group.
[146] Chaput, J. P. et al. (2011)	Healthy subjects N = 22(M); Mean age: 16.7 6 1.1	Significantly higher energy expenditure and significantly increased plasma glucose concentrations during the VG play. No difference in cortisol, ghrelin, and insulin levels.

Beck Depressive Inventory: BDI; Internet Addiction Test: IAT; Internet Addictive Disorder Diagnostic Criteria: IADDC; Internet Addiction Diagnostic Questionnaire: IADQ; Diagnostic Questionnaire for Internet Addiction: DQIA; Online Game Addiction Scale for Adolescents: OGASA; Pathological Internet use scale: PIUS; Scale for the Assessment of Pathological Computer-Gaming: CVS-S; Korean Internet Game Addiction Scale: IGS; IAS: Internet Addiction Scale; RD: Reward-Dependence scale; Wisconsin Card Sorting Test: WCST; Young Internet Addiction Scale (YIAS).

## Data Availability

Not applicable.
